# Evaluating the integrity of forested riparian buffers over a large area using LiDAR data and Google Earth Engine

**DOI:** 10.1038/s41598-020-69743-z

**Published:** 2020-08-24

**Authors:** Hamdi A. Zurqani, Christopher J. Post, Elena A. Mikhailova, Michael P. Cope, Jeffery S. Allen, Blake A. Lytle

**Affiliations:** 1 Department of Forestry and Environmental Conservation, Clemson University, Clemson, SC 29634 USA; 2 Department of Soil and Water Sciences, University of Tripoli, Tripoli, 13538 Libya; 3 South Carolina Water Resources Center, Clemson University, Pendleton, SC 29670 USA; 4 Soil Health Institute, Morrisville, NC 27560 USA; 5 Clemson Center for Geospatial Technologies, Clemson University, Clemson, SC 29634 USA

**Keywords:** Environmental sciences, Environmental impact

## Abstract

Spatial and temporal changes in land cover have direct impacts on the hydrological cycle and stream quality. Techniques for accurately and efficiently mapping these changes are evolving quickly, and it is important to evaluate how useful these techniques are to address the environmental impact of land cover on riparian buffer areas. The objectives of this study were to: (1) determine the classes and distribution of land cover in the riparian areas of streams; (2) examine the discrepancies within the existing land cover data from National Land Cover Database (NLCD) using high-resolution imagery of the National Agriculture Imagery Program (NAIP) and a LiDAR canopy height model; and (3) develop a technique using LiDAR data to help characterize riparian buffers over large spatial extents. One-meter canopy height models were constructed in a high-throughput computing environment. The machine learning algorithm Support Vector Machine (SVM) was trained to perform supervised land cover classification at a 1-m resolution on the Google Earth Engine (GEE) platform using NAIP imagery and LiDAR-derived canopy height models. This integrated approach to land cover classification provided a substantial improvement in the resolution and accuracy of classifications with F1 Score of each land cover classification ranging from 64.88 to 95.32%. The resulting 1-m land cover map is a highly detailed representation of land cover in the study area. Forests (evergreen and deciduous) and wetlands are by far the dominant land cover classes in riparian zones of the Lower Savannah River Basin, followed by cultivated crops and pasture/hay. Stress from urbanization in the riparian zones appears to be localized. This study demonstrates a method to create accurate high-resolution riparian buffer maps which can be used to improve water management and provide future prospects for improving buffer zones monitoring to assess stream health.

## Introduction

Despite the relatively low spatial extent of riparian buffer areas, they are a major concern for land and water resource managers. Evaluation of the land cover within the riparian buffer areas is critical to protecting water quality^1^. The effects of different land cover at the watershed scale can influence the flow of water and nutrients to water bodies, resulting in impacts to stream water quality^2^ from increased sedimentation, higher nutrient and contaminant concentrations, and changes to hydrological patterns^3^. Land cover mapping is essential to obtain a better understanding of interactions and relationships between human activities and the environment over time. The effect of change in land cover varies by region, geographical location, and spatial scale. Quantifying and assessing land cover is essential to formulating integrated land and water resources management strategies^4,5^. Land cover classification can be used as a proxy to the human footprint, which can result in land degradation and a loss of biodiversity^6^.

Rapid population growth in the Savannah River basin region has had dramatic impacts on the natural land cover^7^. This change is most evident in increasing urbanization and conversion of farmland and forests to urban areas, which may decrease the water supply for human use and increase possible human health threats^7^. Past research indicates that 30% or more impervious area in a watershed degrades stream ecosystems^8^. As urban areas grow and envelop forests and agricultural areas, the impacts on lakes, streams, and rivers can be considerable and permanent if there is no control of stormwater^9^. The presence of forests in riparian buffer areas can preserve stream water quality, protect wetlands and floodplains, limit erosion, and offer recreational opportunities^10^. Forests have the potential to improve watershed health and stream water quality through the reduction in stormwater quantity and pollutant runoff that reaches water bodies^11^.

Over the past decades, human activity has been the primary source of land cover/land use changes in the Savannah River basin^7,12,13^. A management plan for the Georgia portion of the Savannah River basin was developed using remotely sensed data and indicated that the use of high-resolution imagery (e.g., aerial photography, etc.) provided more accurate results to detect land cover changes in the region^14^. Environmental disturbances in the Savannah River Basin has led to a decrease in water bodies, vegetation, loss of harvested agricultural land, and farms^7^. Merem et al.^13^ evaluated the environmental conditions in the Lower Savannah watershed in Georgia and South Carolina using spatial–temporal environmental analysis and reported that the region has experienced widespread pollution as shown with the common presence of toxins in the watershed along with high pumpage of water and environmental declines triggered by many stressors including socio-economic factors. At a smaller spatial scale, it has been observed that the conversion of land from forest and agricultural use to urban and suburban use can lead to the degradation of aquatic ecosystems in small streams in the South Carolina region, with the effects being particularly destructive during the actual land conversion process^15–17^.

The amounts and temporal variations in the delivery of water, sediments, and nutrients from land surfaces to stream channels can be influenced by the land cover type^18^. Human-induced land use changes substantially modify land cover, which alters fluxes of water and sediment through stream channel networks^19^. It is challenging to delineate these channels accurately using satellite image classification because it is difficult to distinguish between the channel and floodplains. These analyses are strongly influenced by the Digital Elevation Model (DEM) resolution and quality^20^. Evaluating the condition of the riparian buffer areas along the stream channels can provide information on water quality^21^. Riparian buffers are typically characterized using the Multi-Resolution Land Cover (MRLC) dataset available in the National Land Cover Database (NLCD), which has full national coverage in 30 m resolution but is only available in specific years^7^.

In recent years, Light Detection and Ranging (LiDAR) data have been used successfully to characterize riparian buffers^22–24^. LiDAR data are used to produce Digital Surface Models (DSM), which represents the topography of objects on the earth. LiDAR technology provides an ideal data source to acquire accurate land cover metrics. In the forested landscape, LiDAR can be used to estimate tree canopy height. High-resolution aerial photos can provide detailed spatial information including texture, color, and shape, as well as certain spectral information^25^, but do not provide topography information about trees and other objects on the ground surface. This lack of height information can be compensated for by using LiDAR data, which contains detailed three-dimensional data, but has limited spectral information. The integration of high-resolution images and LiDAR data provides the data necessary for extracting building and forest metrics.

Remotely sensed techniques for land cover/land use analysis have been examined in many studies^7,26–28^. These studies indicate that using remotely sensed data such as Landsat, SPOT, and the National Agriculture Imagery Program (NAIP) imagery can help characterize land cover over large areas. Jacobsen^29^ states that high spatial resolution maps of land cover are often prohibitively expensive, which limits the research and management of moderate to large spatial areas. The National Agricultural Imagery Program (NAIP) provides high-resolution aerial imagery of the continental United States starting in the year 2000. Their data collection was expanded in 2009 to include capturing 4-band imagery in three-year cycles. Nevertheless, some of the states fund a more frequent acquisition. Hayes et al.^30^ were able to create a high-resolution land cover classification map at one-meter resolution using NAIP imagery with an overall accuracy of 81%. Nagel and Yuan^31^ extracted land cover/land use and impervious surface information over large areas from high-resolution remote sensing data with an overall accuracy of 74% and 95% for the general land cover/land use classification and the impervious surface map, respectively. Zurqani et al.^28^ classified NAIP imagery and achieved an overall accuracy of approximately 90% for mapping urbanization trends in a forested landscape in Upstate South Carolina.

Recent research shows that the rapid improvements in the availability of high-resolution geospatial data will facilitate the mapping of geomorphic drivers and contexts across large regions^7,17,28,32^. Conventional geospatial techniques for mapping and monitoring land cover changes such as deforestation, urban growth, agriculture expansion, and wetland loss necessitate downloading remote sensing data and having an appropriate computing power to classify imagery^7^. This is often costly both in terms of computing infrastructure and time, particularly when analyzing large areas and/or extended time periods. A solution to many of these challenges is Google Earth Engine (GEE), a remote sensing platform, which combines an extensive geospatial data catalog with distributed computing resources in a cloud framework and can analyze big data rapidly, which is critical for studying environmental changes over large areas^7,32^. Google Earth Engine enables users to run algorithms on an extensive archive of georeferenced images and other data within Google's infrastructure. With distributed computing (e.g., GEE, etc.) and more LiDAR data available at no-cost, it is now possible to characterize large spatial extents and resolutions that were not possible using conventional methods^33–35^. This study employs a new approach that improves the characterization of land cover in riparian zones over relatively large spatial extents. The present study uses GEE along with high-resolution NAIP images and publically available LiDAR data sets to investigate the land cover within riparian buffer zones over a large area of the southeastern of the United States.

The objectives of the study were to: (1) determine the classes and the distribution of land cover within the stream riparian areas, (2) examine the discrepancies in the existing National Land Cover Database (NLCD) using high-resolution NAIP imagery and LiDAR data, (3) develop a technique using LiDAR data to help characterize riparian buffers over large spatial extents.

## Materials and methods

### Study area

**Figure 1:**
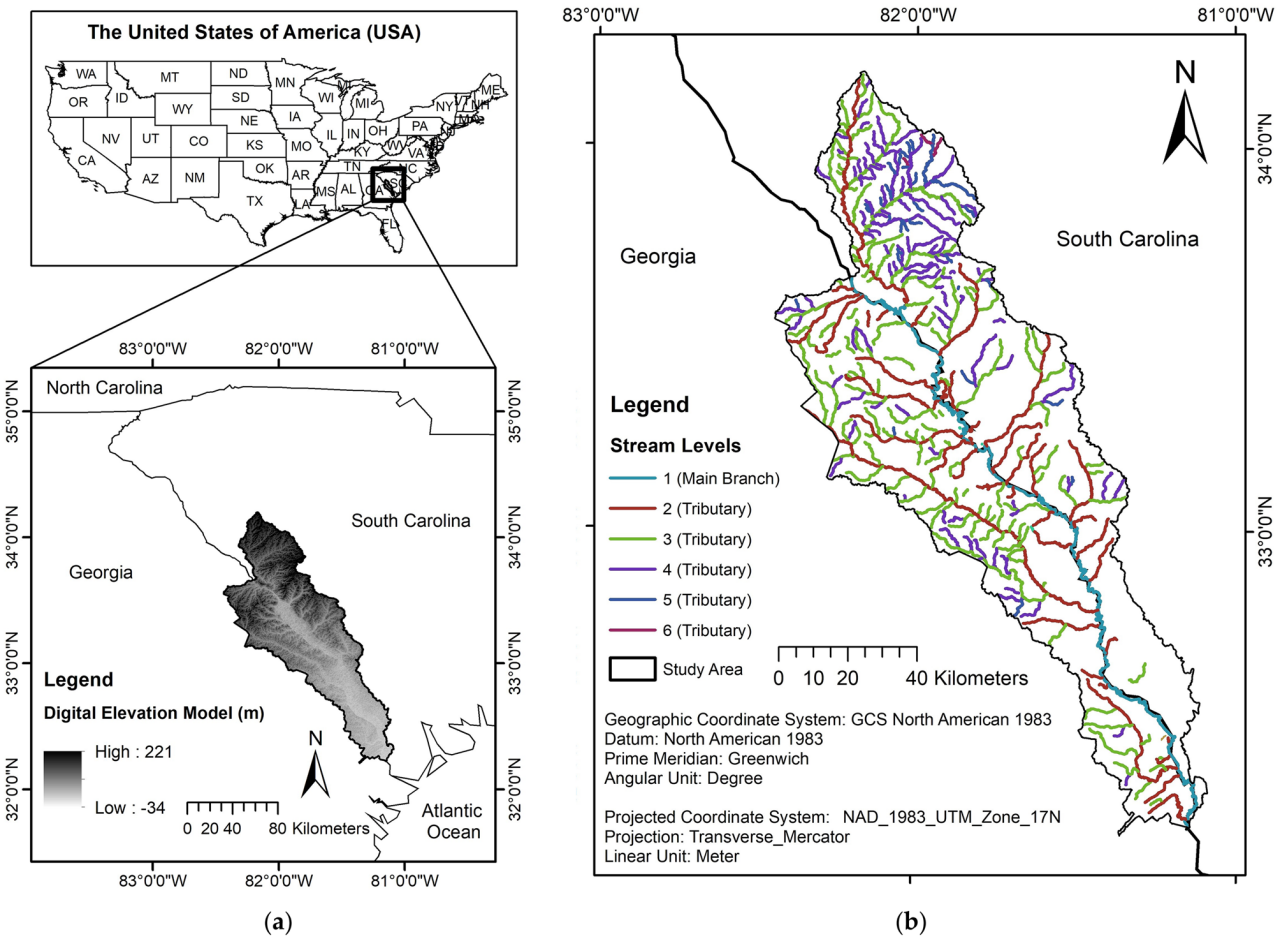
Location of the Savannah River Basin: (**a**) the Digital Elevation Model (DEM), and (**b**) stream channels.

The study area is the lower part of the Savannah River Basin (Fig. 1a,b). It covers approximately 9,880 km^2^ and spans portions of the states of South Carolina and Georgia. The average annual temperature is 18 °C, and the average annual precipitation ranges between 1 and 2 m for the entire basin^14^.

The lower basin of the Savannah watershed (Fig. 1a,b) furnishes water for large urban areas in Georgia and South Carolina. It is an area of high biodiversity and provides habitats for at least nine threatened and endangered species^13^. A variety of different land covers exists in the Savannah River Basin, with much of the riparian area being covered by deciduous forests and wetlands^7^. Most of the overall study area consists of evergreen forests and agriculture areas^7^. Some of the common threats to water quality include the presence of leaky septic tanks and chemical runoffs from farm operations which may cause a serious threat to biodiversity in the region^11,36^. The contamination of water supplies constitutes a growing risk to public health, communities, wildlife, and the ecotourism economy. Approximately 60% of Georgia’s waterways are so highly contaminated that they do not meet the minimum federal criteria for fishing or swimming^13^.

### Data and methods

**Figure 2:**
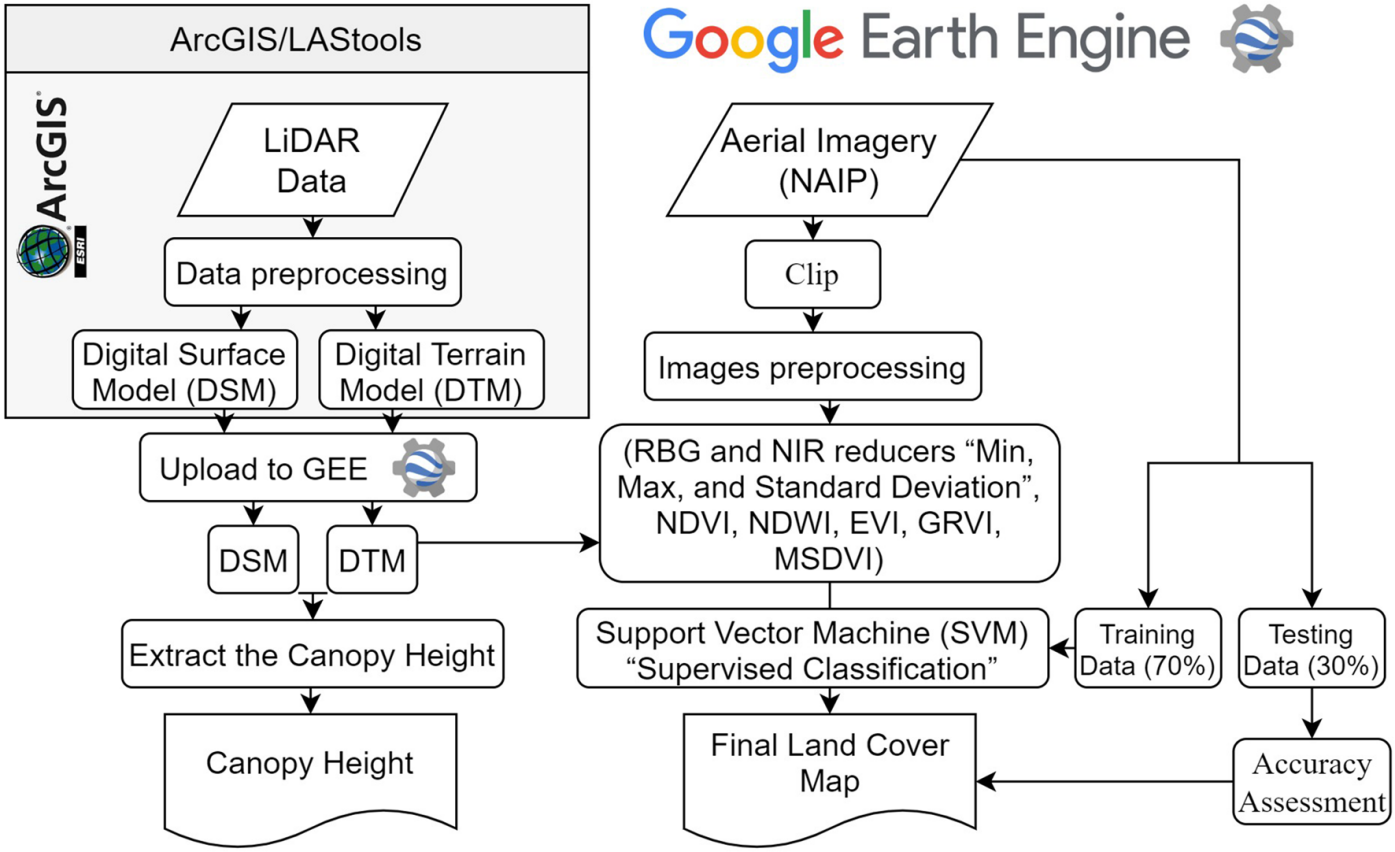
A flow diagram for data processing and the analysis steps.

The land cover classification technique used in this study requires image preprocessing and normalization, as well as a reference dataset to train and evaluate the classification approach. The land cover classification technique was applied and evaluated by developing code in the GEE platform using the supervised classifier algorithm and NAIP imagery for each chosen year. High-resolution aerial imagery was used as a reference dataset for training and validating the classifications. The resulting land cover classification was compared to the NLCD data and the integrity of forested riparian buffers areas was evaluated using NLCD and LiDAR data. The general procedures are summarized in the flowchart illustrated in (Fig. 2).

### Image preprocessing

Google Earth Engine greatly reduces the analysis time by utilizing Google’s distributed computing infrastructure platform (https://earthengine.google.org/). It provides excellent performance in terms of enabling access to remote sensing products through the cloud platform and providing pre-processing to archived data from the US Geological Survey (USGS) collection^7,37^.

The National Agriculture Imagery Program (NAIP) imagery was loaded as an ImageCollection and mosaicked as one single image for the selected date range (Jan 01, 2010, to Dec 01, 2011) (Table 1, Fig. 2). The single composite of the NAIP imagery included the red, green, blue (RGB, ‘visible spectrum’), the near-infrared (NIR) band, and the statistics image neighborhoods of these bands including the minimum (min), maximum (max), and standard deviation (sd) values where the window size and shape specified by “ee.kernel” of the 1 × 1 m neighborhood around the corresponding input pixel. The composite then serves to generate several indices that were derived from spectral band combinations to distinguish features that are more representative of vegetation greenness, such as, the Normalized Difference Vegetation Index (NDVI)^27^, the Enhanced Vegetation Index (EVI)^38^, the Green Ratio Vegetation Index (GRVI)^36^, and the Modified Soil Adjusted Vegetation Index (MSAVI)^39^ to enhance the classification accuracy. The Normalized Difference Water Index (NDWI)^40^ was used to better distinguish between water and vegetated areas^27^. All of these indices were calculated for each image and stacked for later classification (Fig. 2). These indices are expressed in the following Eqs. (1–5):

1 $$NDVI =\frac{\left(NIR-Red\right)}{\left(NIR+Red\right)}$$

2 $$EVI=G\frac{\left(NIR-R\right)}{\left(NIR+C1*Red-C2*Blue+L\right)} *100$$

3 $$GRVI= \frac{NIR}{Green} *100$$

4 $$MSAVI=\frac{(2*NIR+1- \sqrt{{(2*NIR+1)}^{2}-8*(NIR-Red)}}{2}*100$$

5 $$ NDWI = \frac{{\left( {Green - NIR} \right)}}{{\left( {Green + NIR} \right)}} $$

where Red, Green, Blue and NIR are the NAIP imagery bands. The coefficients adopted in the MODIS-EVI algorithm are; L = 1, C1 = 6, C2 = 7.5, and G (gain factor) = 2.5.

**Table 1:** Data sources and description.

Data layer	Source	Resolution	Date
Watershed Boundary Dataset (WBD), Streams	US Department of the Interior, US Geological Survey	Scale 1:24,000	2016
National Hydrography Dataset Plus (NHDPlus)	US Department of the Interior, US Geological Survey	Scale 1:24,000	2012
The National Agriculture Imagery Program (NAIP)	Google earth engine (GEE) data provided by US Department of Agriculture (USDA) Farm Service Agency	0.6 m	2010–2011
LiDAR data	County offices, Clemson University, National Oceanic and Atmospheric Administration (NOAA)	1 m	2010–2011
NLCD: USGS National Land Cover Database	Google earth engine (GEE) data provided by U.S. Geological Survey (USGS)	30 m	2011

### LiDAR data processing methods and canopy height calculation

One-meter resolution Digital Terrain Model (DTM), and Digital Surface Model (DSM) raster data were produced for each county using LiDAR point clouds in a High Throughput Computing (HTC) environment consisting of 20–30 desktop computers in the Clemson University Center for Geospatial Technologies. LiDAR data were compiled in coordination with numerous county representatives or downloaded from the NOAA Coastal Topographic LiDAR repository (https://coast.noaa.gov/) and stored on 4-terabyte hard drives.

Custom Python scripts were used to automate LiDAR processing, which included sub-processing LASTools, based on triangular irregular network (TIN) interpolation^41^ and ArcGIS 10.6 software^42^ in order to construct DTM and DSM products in a piece-wise fashion. For each tile (approximately 1 km^2^), the Python scripts collect available LAS point clouds from neighboring tiles, execute the blast2dem script in LASTools using a 200-m buffer radius, and then execute ArcGIS scripts in order to mosaic and clip the DSM and DTM of the center or target tile. HTCondor software^43^, the specific HTC software, was used to distribute jobs across many computers. HTCondor returned the finished DSM and DTM tiles and individual tiles from each job were mosaicked into a final DSM and DTM at the county level.

Finally, the DTM and DSM layers were uploaded to the GEE platform and used to generate the canopy height. The canopy height represents the height or distance between the ground and the top of the objects above the ground and it was calculated by subtracting the DTM from the DSM.

### Land cover categories and reference data

The Savannah River basin is a heterogeneous landscape comprised of a diverse mix of aquatic and terrestrial habitats^12^ that are challenging to classify from satellite imagery. It is possible to reliably define eleven land cover classes by utilizing the 0.6-m resolution NAIP aerial imagery^7^ and the NLCD land cover description^44^ (Fig. 4a). The following land cover classes were identified: (1) open water with lakes, rivers and water bodies, (2) low-medium intensity urban with paved roads/concrete structures and limited buildings, (3) high intensity urban with infrastructure indicating areas with dense human population, (4) barren land, (5) deciduous forest, (6) coniferous forest, (7) shrub/scrub, (8) grassland/herbaceous, (9) pasture/hay, (10) cultivated crops, and (11) wetlands with woody and emergent herbaceous wetlands.

Ground reference data (ground truth) plays a key role in supervised image classification. The number of reference data sets used is also a critical factor in this step^28^. Visual interpretations were used to produce a total of 4,390 reference points for the year 2011 using NAIP imagery with a no less than 250 reference points per land cover category. Each point was buffered by one meter to enhance the classification results and ensure an objective identification of the reference data and the NAIP imagery data. The reference dataset was split randomly into a training dataset set consisting of 70% of the observations and a testing dataset with the remaining 30% of the observations^7^. The training dataset was used to train the supervised classifier algorithm, while the testing data set was used to assess the accuracy of the resulting land cover classification map.

### Support vector machine classifier

A wide variety of classification algorithms have been used to classify and map land cover from remotely sensed data^45,46^. Supervised machine learning classifiers, such as Support Vector Machine (SVM), Regression Trees (CART), and Random Forest (RF), are increasingly used to classify remotely sensed data^47^.

The SVM classifier has been effective in producing high classification accuracy using high-resolution imagery. The SVM classifier relies exclusively on the training samples that are closest in feature space to the optimal boundary between the classes^48–50^. Support Vector Machine’s are essentially binary classifiers; however, they can be adapted to handle the multiple classification tasks common in remote sensing studies^49–54^. The performance of SVM’s has been shown to be superior to the traditional pattern classifiers (Linear, Quadratic, Fisher Linear Discriminant, Nearest-Neighbor) as well as more modern techniques such as Radial Basis Function (RBF) classifiers and large ensemble-RBF network^55^. Adam et al.^52^ found that SVM and RF classifiers performed equally well in terms of accuracy. In this study, a comparison was implemented between commonly used classifies in the GEE platform including; Decision Tree (DT), Random Forest (RF), and Support Vector Machin (SVM). In this comparison, the SVM classifier achieves the best visual accuracy in the vegetation class than other classifiers, and also performs satisfactorily to the building and road classes. The SVM classifier algorithm was applied to obtain the land cover classification map for each chosen year of the NAIP imagery. The SVM functions by nonlinearly projecting the training data in the input space to a feature space of higher (infinite) dimension using a kernel function^51^. This results in a linearly separable dataset that can be separated by a linear classifier. This process enables the classification of remote sensing datasets which are usually nonlinearly separable in the input space^51^. Finally, following Yang et al.^56^ a procedure to correct mapping errors by a hand-editing process was applied. In some areas, hand-editing was used to correct misclassified pixels (e.g., impervious, surface reflection in some water areas, etc.).

### Accuracy assessment

Many factors affect the accuracy of image classification. Accuracy assessments are useful and effective techniques to determine how well the classification process accomplished the study objectives^57–59^. The accuracy assessment process allows a comparison between certain pixel values in a raster layer and the reference pixels for which the class is known. The produced land cover classification map was validated using high-resolution imagery (NAIP) (0.6-m resolution). Approximately 1,320 of the reference data points were used in this validation process with no less than 50 reference points per land cover category. The confusion matrix of land cover maps was calculated to evaluate the accuracy of the results using the producer’s accuracy, user’s accuracy, the overall accuracy, Kappa statistics which reflect the difference between actual agreement and the agreement expected by chance as shown in Eq. (6), and F1 score which shows how good the classifier is in the context of both producer’s and user’s by weighting the average of producer’s and user’s ^7,28^ as shown in Eq. (7):

6 $$Kappa\;statistics =\frac{observed\;accuracy - agreement\;chance}{1-agreement\;chance}$$

7 $$ F1\;score = \frac{2}{{\frac{1}{{{\text {producer}}'{\text {s}}}} + \frac{1}{{{\text {user}}'{\text {s}}}}}} = 2*\frac{{{\text {user}}'{\text {s}}* {\text {producer}}'{\text {s}}}}{{{\text {user}}'{\text {s}} + {\text {producer}}'{\text {s}}}}$$

The DTM was used to distinguish between the wetlands and another type of land cover classes in a forested watershed^7,58,60^. The accuracy assessment of the forested cover (tree vs. non-tree) based on the LiDAR canopy height, NAIP classified imagery, and NLCD data was evaluated using 500 points of field observation data that were randomly selected within the stream riparian areas. The results were evaluated using three indicators, i.e., the producer’s accuracy, user’s accuracy, and the F-score.

### Quantifying land cover classes in riparian zones

Buffers were constructed around the stream network in the NHDPlus database (Table 1) at distances of 50, 100, 150, 200, 250, 300, 350, 400, 450, 500, and 550 m. These buffer levels were further categorized using the Strahloer Stream Order level feature in the NHDPlus data. The area of each land cover classification within each buffer distance and stream order level was quantified.

## Results

### Spectral behavior of land cover classes

**Figure 3:**
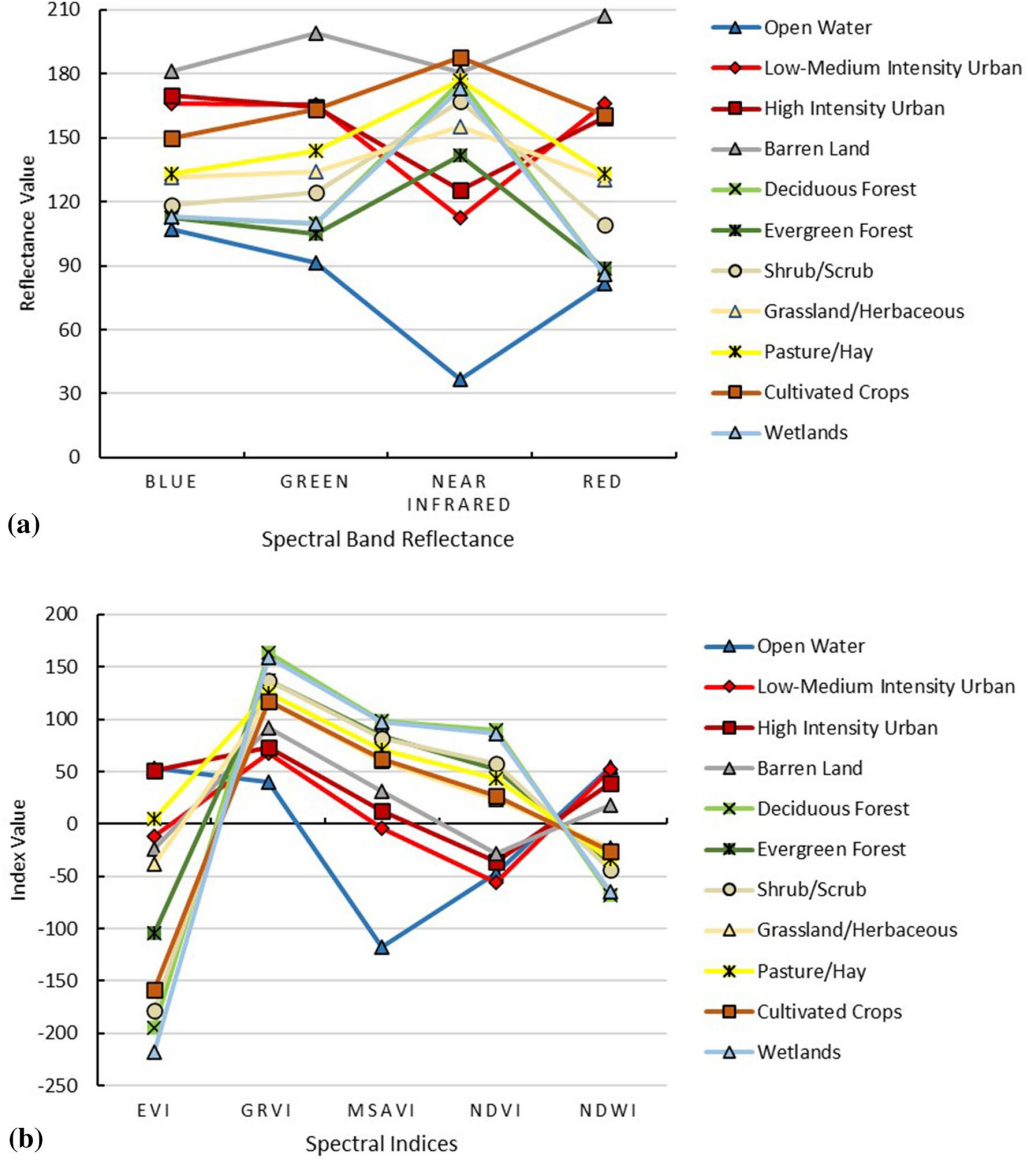
The spectral values of each land cover type in the study area: (**a**) spectral bands reflectance (*R* Red, *G* Green, *B* Blue, *NIR* Near Infrared), and (**b**) indices spectral (*NDVI* Normalized Difference Vegetation Index, *EVI* Enhanced Vegetation Index, *GRVI* Green–Red Vegetation Index, *MSAVI* Modified Soil-Adjusted Vegetation Index, *NDWI* Normalized Difference Water Index).

Spectral resolution refers to the number of bands of data a sensor provides and which part of the electromagnetic spectrum they capture. The spectral signature values of the twelve land cover classes are presented in Fig. 3a,b. The spectral signature values of the actual NAIP imagery bands reflectance (R: Red, G: Green, B: Blue, and NIR: Near Infrared) are shown in Fig. 3a. Generally, the red and NIR parts of the spectrum are most important for vegetation classification. The red band was most useful for distinguishing between the vegetation classes except for the evergreen forest where it was confused with the water spectra. However, the water presented a typical behavior with low reflectance in NIR values. The reflectance of bare land was visible in both the green and the red bands. The urban areas were well separated in the blue spectra.

The results of the spectral indices, which include NDVI: Normalized Difference Vegetation Index, EVI: Enhanced Vegetation Index, GRVI: Green–Red Vegetation Index, MSAVI: Modified Soil-Adjusted Vegetation Index, and NDWI: Normalized Difference Water Index are shown in Fig. 3b. The vegetation classes were easy to distinguish in the EVI, while water was much easier to identify in the MSAVI.

### Accuracy of support vector machine classifier

**Figure 4:**
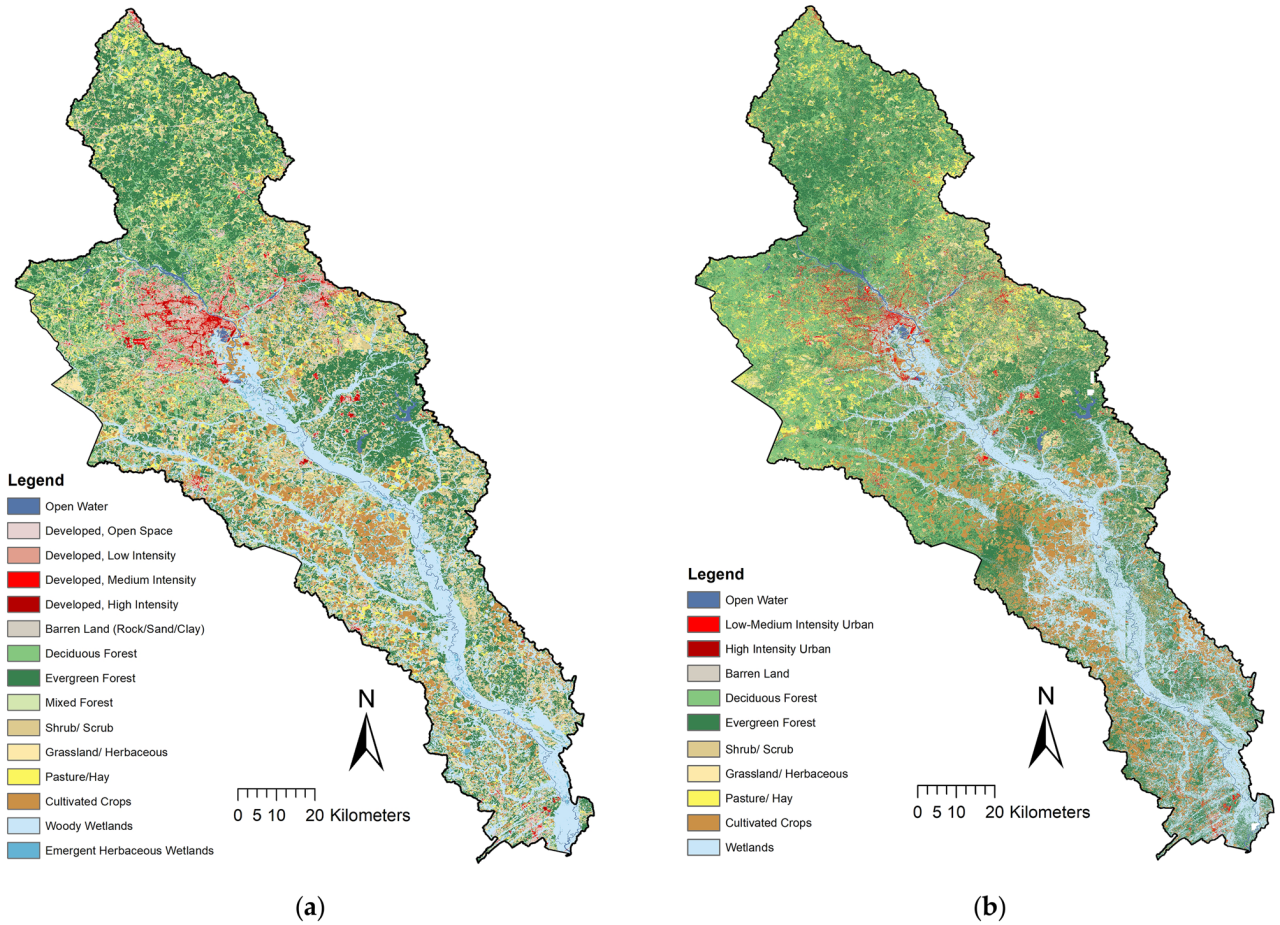
Land cover map: (**a**) the National Land Cover Database (NLCD), and (**b**) high resolution land cover map derived from the NAIP imagery.

The final land cover map used for land cover assessment is shown in Fig. 4b, and producer’s and user’s accuracy of the classification using the SVM algorithm are listed in Table 2. The overall accuracy was 77.65%, while user’s accuracies of each land cover classification range between 55.61% (evergreen forest) and 96.72% (high intensity urban).

**Table 2:** The percentage of producer and user accuracy, F1 score, overall accuracy, and kappa statistic for land cover classification.

No	Types	User's Accuracy	Producer's accuracy	F1 Score
1	Open water	91.80	99.11	95.32
2	Low-medium intensity urban	92.45	87.50	89.91
3	High intensity urban	96.72	76.62	85.50
4	Barren land	89.47	81.92	85.53
5	Deciduous forest	69.18	75.86	72.36
6	Evergreen forest	55.61	77.85	64.88
7	Shrub/scrub	80.20	58.77	67.84
8	Grassland/herbaceous	75.96	59.39	66.67
9	Pasture/hay	84.33	74.46	79.09
10	Cultivated crops	74.62	87.71	80.64
11	Wetlands	78.40	81.66	79.99
	Overall accuracy	77.65		
	Kappa coefficient	75.30		

This result confirms the finding of Nagel and Yuan^31^ who created a high-resolution land cover and impervious surface map in the Twin Cities Metropolitan area using NAIP imagery where they achieved an overall accuracy of 74% and 95% for the general land cover/land use classification and the impervious surface map, respectively. The results of this study show that most of the land cover classes were adequately mapped, except some areas of the shrub/scrub and grassland/herbaceous. Zurqani et al.^7^ explained this classification errors due to the pixel’s similarities between shrub/scrub and grassland/herbaceous and regrowth forests. The Digital Terrain Model (DTM) layer was useful to distinguish between the wetlands and the other land cover classes, such as the deciduous forest areas.

### Distribution of land cover classes

The land cover classification map for the entire study area was created using supervised classification Support Vector Machine (SVM) algorithm with NAIP imagery via GEE for the year 2010/2011 in a total of eleven common land cover categories (Table 2, Fig. 4b).

The distribution of individual class areas is summarized in Table 3. The high-resolution one-meter land cover map derived from NAIP imagery is shown in Fig. 4b. At a larger scale region, the detailed land cover features such as residential areas, local roads and streets, and small water bodies can clearly be identified in this high-resolution classification map. This was followed by low-medium intensity urban 185.30 km^2^, high intensity urban 38.85 km^2^, barren land 122.37 km^2^, and an open water area of approximately 168.85 km^2^. The vegetation classes were classified as the largest land cover classes in the area followed by the deciduous forest 1910.04 km^2^, evergreen forest 3,101.39 km^2^, shrub/scrub 527.60 km^2^, grassland/herbaceous 724.55 km^2^, pasture/hay 468.57 km^2^, cultivated crops 823.38 km^2^, and wetlands 1809.08 km^2^ (Table 3).

**Table 3:** The distribution of land cover classes based on NAIP classification imagery.

No	Types	Area (km^2^)	(%)
1	Open water	168.85	1.71
2	Low-medium intensity urban	185.30	1.88
3	High intensity urban	38.85	0.39
4	Barren land	122.37	1.24
5	Deciduous forest	1910.04	19.33
6	Evergreen forest	3,101.39	31.39
7	Shrub/scrub	527.60	5.34
8	Grassland/herbaceous	724.55	7.33
9	Pasture/hay	468.57	4.74
10	Cultivated crops	823.38	8.33
11	Wetlands	1809.08	18.31
	Overall total	9,880.00	100.00

### Classes and distribution of land cover within riparian areas

**Figure 5:**
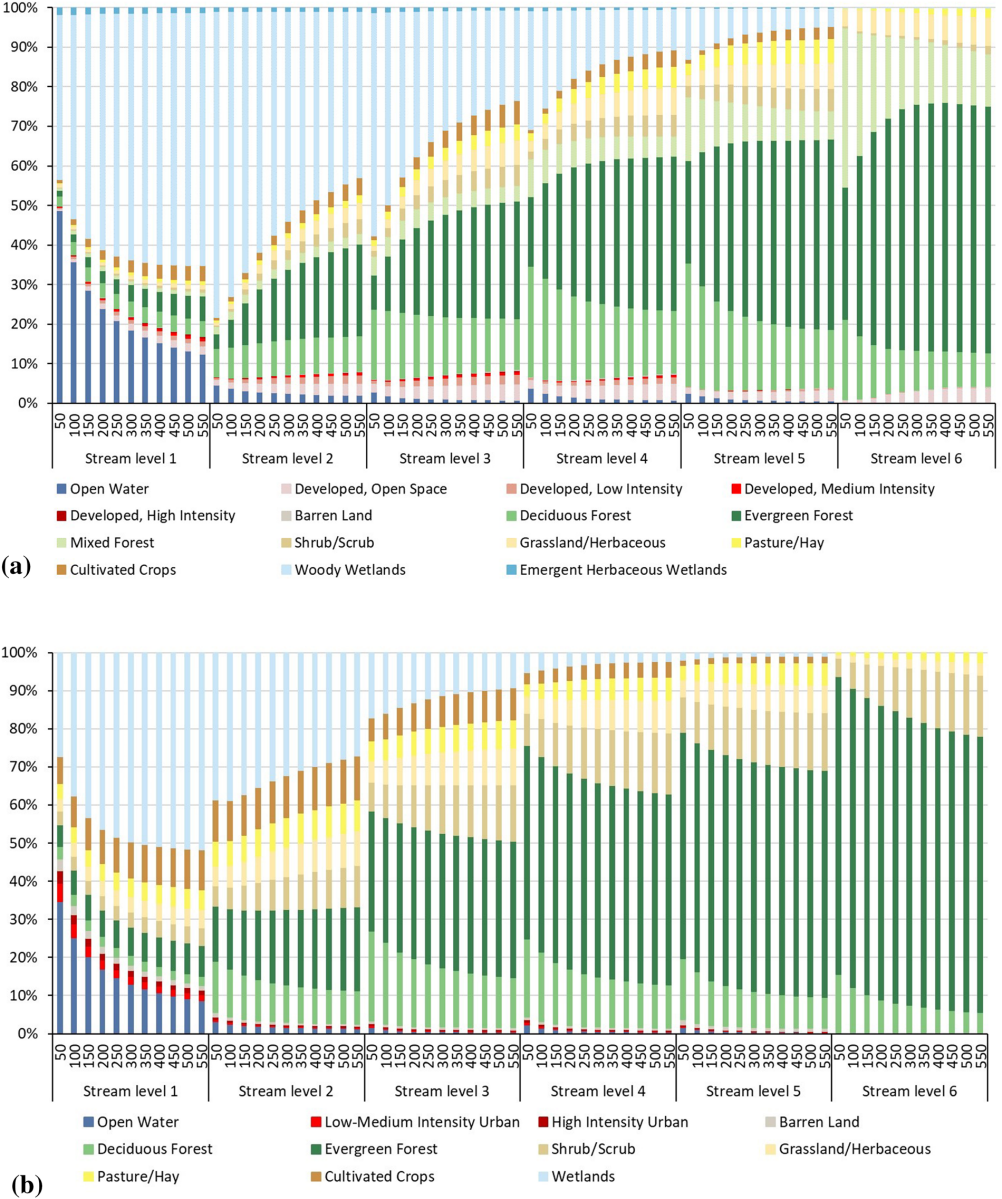
The classes and the distribution of land cover in the stream’s riparian areas from: (**a**) NLCD data, and (**b**) NAIP classified imagery.

**Figure 6:**
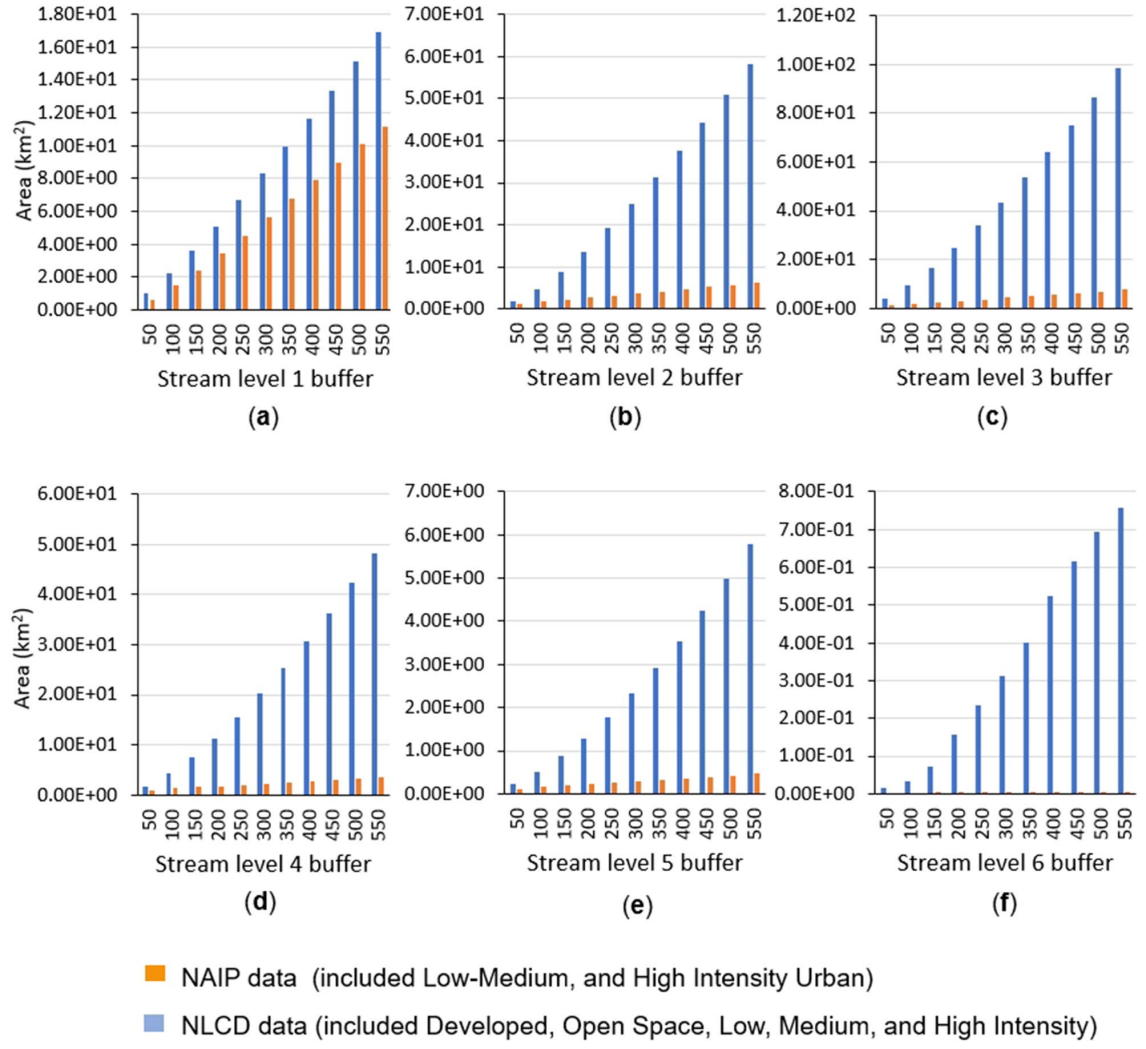
Impervious surfaces within buffers areas using classified NAIP, and NLCD data: (**a**) within level 1 (main branch), (**b**) level 2 (tributary), (**c**) level 3 (tributary), (**d**) level 4 (tributary), (**e**) level 5 (tributary), and (**f**) level 6 (tributary).

The one-meter land cover map derived from NAIP was compared to the 30 m resolution 2011 NLCD. Data were extracted within the stream riparian areas for different buffer zones (50, 100, 150, 200, 250, 300, 350, 400, 450, 500, and 550 m (Fig. 5a,b). In Fig. 5a, a key difference between NLCD classes and the NAIP classifications can be clearly observed. Most notably, NLCD appears to underestimate forest cover within stream buffer 50, 100, 150, 200, and 250 m compared to the NAIP classifications at all buffer distances and stream order levels. A comparison of the total impervious surface areas within the buffers between the classified NAIP and NLCD data is shown in Fig. 6a–f. The results showed that the total impervious surface areas that were extracted from the NLCD data exceeded the results from the classified NAIP classification at all of the streams levels. In particular, this variation is most evident at the buffer zones of stream levels 2, 3, 4, 5, and 6 (Fig. 6a–f).

### Evaluating the integrity of forested riparian buffer areas

**Figure 7:**
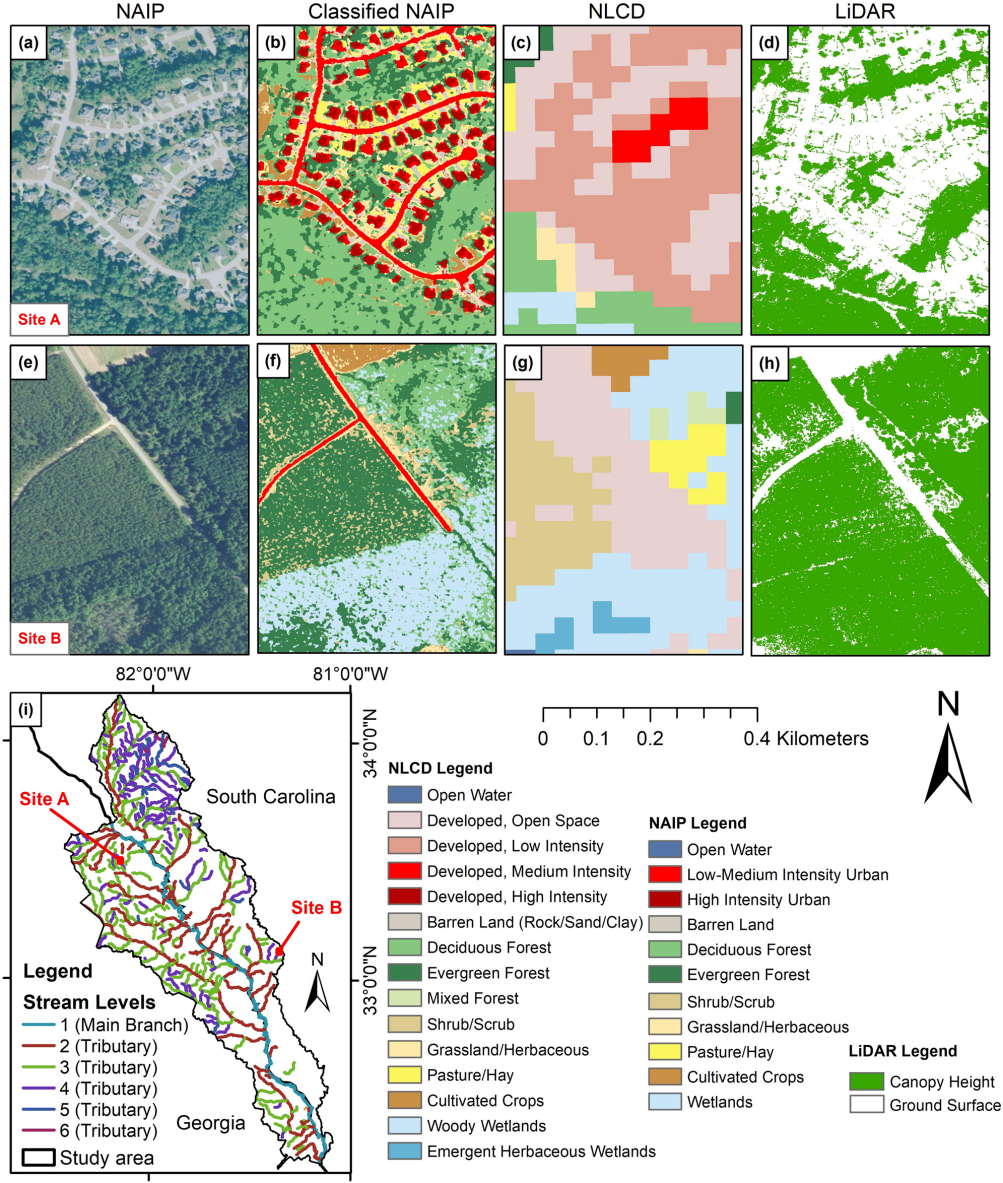
Selected examples of land cover types within the stream’s riparian areas: (**a**) NAIP imagery of site A, (**b**) classified NAIP imagery of site A, (**c**) NLCD data of site A, (**d**) LiDAR (Canopy Height) of site A, (**e**) NAIP imagery of site B, (**f**) classified NAIP imagery of site B, (**g**) NLCD data of site B, (**h**) LiDAR (Canopy Height) of site B, and (**i**) the location of the sites A and B.

In order to evaluate the discrepancies between the forested cover classifications within the riparian buffer areas at different stream levels in both NLCD and classified NAIP imagery, the results were compared to the canopy height (1-m resolution) that derived from LiDAR data. Results of the accuracy assessment (Table 4) show that forest canopy classification using the NAIP classified aerial imagery and the canopy height derived from LiDAR data had the highest accuracies with F-score of 98.85% and 98.53% respectively. In comparison, the NLCD land cover product had less accuracy with an F-score of 86.28%. This difference in accuracy assessment can be explained by the large difference in the forest cover visible in the riparian buffer areas (Fig. 7a–i). Additionally, the difference in spatial resolution between the three types of spatial data can also contribute to this variation in error.

**Table 4:** Accuracy assessments of the forest canopy classification results from the NLCD data, NAIP classified imagery, and the canopy height derived from LiDAR data within stream riparian areas.

Accuracy indicators	NAIP classified imagery^a^	NLCD data^b^	LiDAR data
Producer’s accuracy	97.73	75.87	97.11
User’s accuracy	100.00	100.00	100.00
F-score	98.85	86.28	98.53

^a^NAIP classified imagery (included deciduous and evergreen forests, and wetlands).

^b^NLCD data (included deciduous, evergreen, and mixed forests, and woody wetlands).

**Figure 8:**
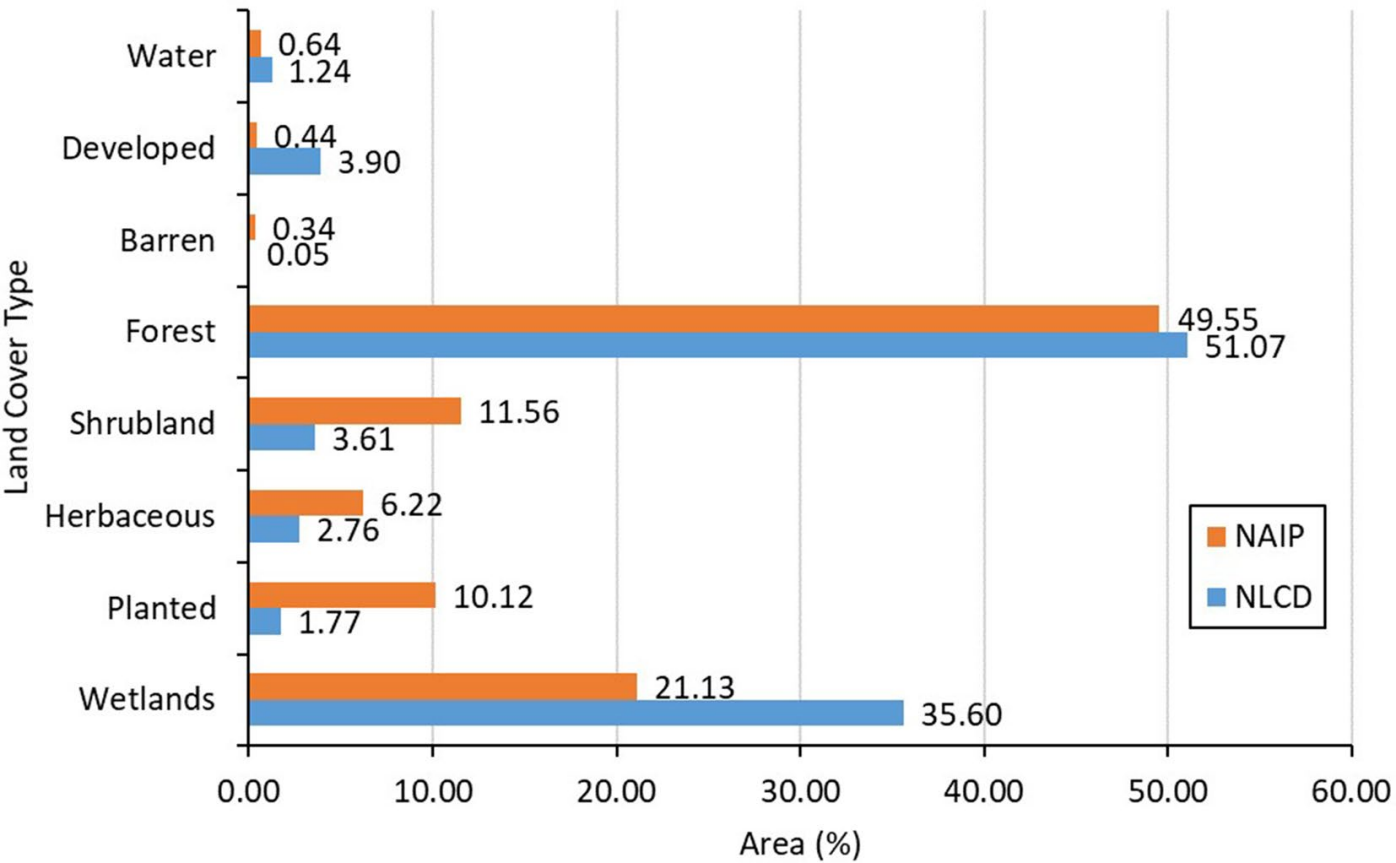
The percentage of the masked areas of both land cover layers NLCD data and NAIP classified imagery with the Canopy Height derived from LiDAR data within stream riparian areas.

The land cover classification using NAIP imagery and LiDAR provide a more detailed and accurate accounting of land cover area as shown in (Fig. 7a–i). For example, the SVM using high-resolution NAIP imagery is able to detect pockets of forest within developed areas while the NLCD provides a more generalized classification. Though both the classified NAIP and NLCD classifications show that forest and wetlands are the dominant land cover classes in the riparian areas of the Lower Savannah River Basin, the higher resolution of NAIP and the accuracy of the SVM resulted both in different proportions of land cover classes and a higher resolution classification (Figs. 7, 8).

### The variations of land cover types within canopy height areas

The land cover types within stream riparian areas from NLCD data and classified NAIP imagery were masked using the canopy height derived from the LiDAR data to estimate the land cover areas corresponding to canopy heights above ground elevation. A large difference in the total land cover in this area can be seen in Table 5 and Fig. 8. These results in Table 5 and Fig. 8 show that the total impervious surface was much higher in NLCD data (101.91 km^2^, 3.90%) compared to NAIP classified imagery (11.57 km^2^, 0.44%). While the total forested areas from NAIP classified imagery (1,295.48 km^2^, 49.55%) were smaller than NLCD data (1,335.24 km^2^, 51.07%). Apart from these variations, differences were also observed in the other land cover types where the total areas of shrubland, herbaceous and planted from the NAIP classified imagery were higher than the NLCD data with about (302.21 km^2^, 11.56%), (162.53 km^2^, 6.22%), and (264.53 km^2^, 10.12%) respectively. While the total wetlands from NAIP classified imagery (552.55 km^2^, 21.13%) were smaller than NLCD data (930.69 km^2^, 35.60%).

**Table 5:** The masked areas of both land cover layers NLCD data and NAIP classified imagery with the canopy height derived from LiDAR data within stream riparian areas.

Land cover	NLCD data	Area (km^2^)	NAIP classified imagery	Area (km^2^)
Water	Open water	32.47	Open water	16.85
Developed	Developed, open space^a^	70.65	Low-medium intensity urban	5.64
	Developed, low intensity^a^	24.88		
	Developed, medium intensity^a^	4.45		
	Developed, high intensity^a^	1.94	High intensity urban	5.93
	Total	101.91		11.57
Barren	Barren land	1.33	Barren land	8.84
Forest	Deciduous forest	344.15	Deciduous forest	303.27
	Evergreen forest	875.80	Evergreen forest	992.21
	Mixed forest	115.29	–	–
	Total	1,335.24		1,295.48
Shrubland	Shrub/scrub	94.50	Shrub/scrub	302.21
Herbaceous	Grassland/herbaceous	72.19	Grassland/herbaceous	162.53
Planted	Pasture/hay	22.22	Pasture/hay	116.82
	Cultivated crops	24.00	Cultivated crops	147.70
	Total	46.23		264.53
Wetlands	Woody wetlands	922.46	Wetlands	552.55
	Emergent herbaceous wetlands	8.22	–	–
	Total	930.69		552.55

^a^Distribution of percentage imperviousness among developed land-cover types (Open space—<20%; low intensity—20−49%; medium density—50–79%; and high density—80–100%) in NLCD data.

## Discussion

Advantages and limitations of the approach that this land cover classification approach for riparian areas uses the Support Vector Machine (SVM) supervised classification algorithm with NAIP imagery data and includes a number of indices within the GEE platform (Fig. 2). The (SVM) algorithm adequately classifies the heterogeneous land cover in the lower part of the Savannah River basin and produces reliable land cover results with the ability to differentiate disparate types of land cover^53^. Rudrapal and Subhedar^54^ employed the SVM algorithm for automated classification of various land cover types using hyperspectral imagery and successfully achieved an overall accuracy of more than 90% almost in all cases of land cover. This approach also utilized LiDAR data to produce a canopy height, which helped to identify forest cover in the study area and evaluate the integrity of forested landscape within the riparian buffer areas.

The GEE platform supports high-speed data analysis using processing functions for large spatial extents while also supporting the use of algorithms that pool data from multiple years, sensors, and models^7^. The approach presented here results in accurate land cover classifications and easily be repeated as new remote sensing layers are ingested into the GEE platform, which will help highlight the wide variety of earth surface disturbances over time.

A limitation of utilizing this approach is that NAIP imagery and LiDAR data are not available for all years at all locations. The availability of the NAIP imagery is based upon available funding and the Farm Service Agency (FSA) imagery acquisition cycle, where it began a three-year cycle in 2009. LiDAR data availability can be also vary depending on the location within the United States. For instance, the National Oceanic and Atmospheric Administration (NOAA) provides LiDAR data for only a few states in the USA. LiDAR data products produced and used in this study also require substantial computational infrastructure and storage capacity. Infrastructure similar to GEE is needed for storage and derivation of LiDAR data products in order to make the approach used here more widely applicable.

The lower Savannah Sub-basin is a large area of 2.5 million acres. Mapping this large spatial extent of high-level vegetation and urban details using high-resolution imagery provides a valuable addition to land cover mapping. The results of the land cover classification approach conducted in this study concur with the large-scale impact of expanding forest coverage in the region. A key advantage to the NAIP-derived land cover map was the fine spatial resolution that allowed the very-local-scale analysis of riparian buffer areas. The results confirmed that the rapid improvements in the availability of high-resolution geospatial data with distributed computing such as GEE can facilitate the mapping of geomorphic drivers and contexts across large regions. These findings provide evidence that may help facilitate future land cover and land use planning, management, and decision-making in the watershed area. In addition, this assessment of land cover within the riparian areas may also help to explain and respond effectively to emerging environmental risks in the region.

The canopy height derived from the LiDAR data illustrates that there is a need for using high-resolution data to evaluate land cover within the riparian buffer areas. In this study, both results from the NAIP classified imagery and LiDAR data provided reliable accuracies to assess the integrity of forested riparian buffers over the study area. LiDAR canopy height adds useful information for land cover classification, especially at high spatial resolution. For example, canopy height can help the classifier algorithm distinguish between different levels of vegetation height, which would help differentiate between wetlands and forests. Similarly, it could help the classifier distinguish between forests of different ages. Land cover classification can and should be included in these efforts, along with LiDAR data products.

Access to computational infrastructure and LiDAR data is a barrier to the wider adoption of this type of classification approach. High-resolution LiDAR data require substantial storage capacity and computing power to process into data products for large spatial extents. Using high-resolution LiDAR data products such as DSM and DTM requires significant storage and processing infrastructure. The custom Python scripts and workflow used in this study can be used to streamline the process. These scripts build a spatially indexed cursor table that is used to select and “package” LiDAR data files for processing into data products. This increases the speed of selecting data and allows jobs to be distributed out across many computers using the HTCondor software. The workflow is, therefore, more efficient and reliable than trying to perform these tasks for a large area from a single work station computer. Moving forward with tools like Python, LAStools, ArcGIS, and HTCondor, there is still considerable need for centralized repositories where LiDAR point cloud data and data products can be archived for wider access.

## Conclusions

Characterizing riparian buffer conditions is a critical first step in environmentally sound resource management and planning for maintaining water quality. This study demonstrates that the availability of historical remotely sensed data as well as the new geospatial technology of GEE represents a significant improvement for monitoring and evaluating land cover over large areas. In this study, a regional scale analysis was successfully developed using high-resolution imagery and determines the classes and distribution of land cover in the lower part of the Savannah River Basin and evaluated the integrity of forested landscape within the riparian buffer areas. Multiple-layers were used, including the original four bands RGB and NIR, NDVI, EVI, GRVI, MSAVI, and NDWI, which provided reliable results in classifying six general land cover types. These vegetation indices were very useful to enhance the classification result, and MSAVI was the optimal index to separate water bodies from other types of land cover.

The results showed that the NAIP classified imagery provides more accurate results to identify and quantify the land cover classes than the NLCD data, especially near urban areas. Both results from the NAIP classified imagery and LiDAR data provided reliable estimates of the integrity of forested riparian buffers over the study area. It is also shown that NAIP imagery and LiDAR data can be used to accurately map the vegetation width, height, and canopy cover within the riparian buffer over wide areas to support ecological-based management.

The proposed methodology can be used to accurately quantify the land cover and canopy height within the riparian buffer width at the spatial extent and resolutions that were not possible using conventional methods. It is also highlighted that the open-access imagery and efficient geospatial analysis GEE provides a powerful and reliable methodology to remotely monitor riparian area integrity. The availability of this geospatial platform at no cost to non-commercial users and the advantage of this proposed approach can be useful for developing detailed land cover maps. This type of information facilitates research and management in maintaining riparian areas with the highest ecological integrity. Furthermore, it provides useful knowledge in understanding pollution sources of the river water quality, which provides information to policymakers to help sustainably manage land cover. The limitation of this approach is that NAIP imagery, and other sources of high-resolution aerial imagery are not typically available on a yearly basis. In future research, continuous monitoring of land use change is needed to better understand its impact in the region and which returns more effective management strategies.
